# P-519. Long-acting Injectable PrEP Uptake and Persistence in a Sexual Health Clinic

**DOI:** 10.1093/ofid/ofae631.718

**Published:** 2025-01-29

**Authors:** Nicholas Francisco Massanet, Jonathan George, Veena Jajoo, Kerri Bevard, Sheila Forstell, Maritza Acosta, Marcia Bookins, Charurut Somboonwit, Jacqueline E Sherbuk

**Affiliations:** University of South Florida, Valrico, Florida; Hillsborough Health Department, Tampa, Florida; University of South Florida, Valrico, Florida; Hillsborough Health Department, Tampa, Florida; Hillsborough Health Department, Tampa, Florida; Hillsborough Health Department, Tampa, Florida; Hillsborough Health Department, Tampa, Florida; University of South Florida Morsani College of Medicine, Tampa, Florida; University of South Florida, Valrico, Florida

## Abstract

**Background:**

HIV pre-exposure prophylaxis (PrEP), a powerful tool for HIV prevention, has traditionally been taken as a daily oral medication. Long-acting injectable (LAI) cabotegravir is superior to daily oral PrEP for HIV prevention and was approved for use as LAI-PrEP in 2021. We aimed to evaluate the uptake of LAI-PrEP and six-month LAI-PrEP persistence in a sexual health clinic.

Participant characteristics (N=36)

**Figure:**
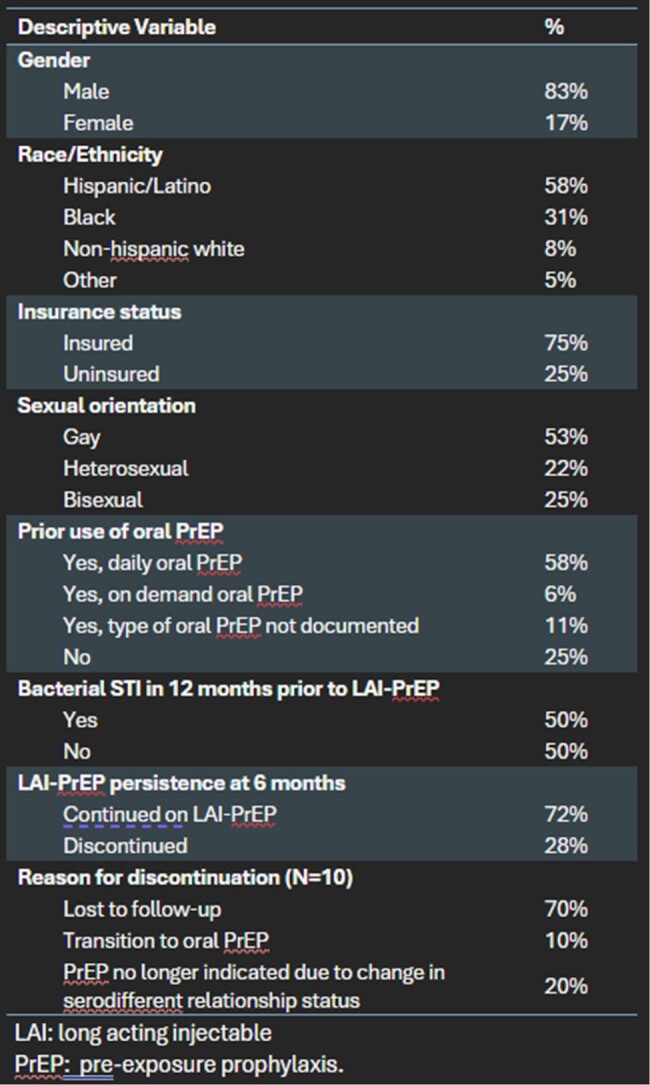

LAI: Long acting injectable

PrEP: Pre-exposure prophylaxis

**Methods:**

In February 2023, an LAI-PrEP pilot was implemented in a county sexual health clinic serving a diverse patient population in a designated ending the HIV epidemic in the South. Through state funding, patients at risk of HIV through sexual transmission were eligible to receive initial doses of LAI-PrEP at no cost and same-day initiation was encouraged. We evaluated the first 12 months of implementation. The primary outcome was LAI-PrEP persistence at 6 months. Statistical analyses were performed (SPSS version 29) using chi-square test for categorical variables with a significance level of 0.05.

**Figure:**
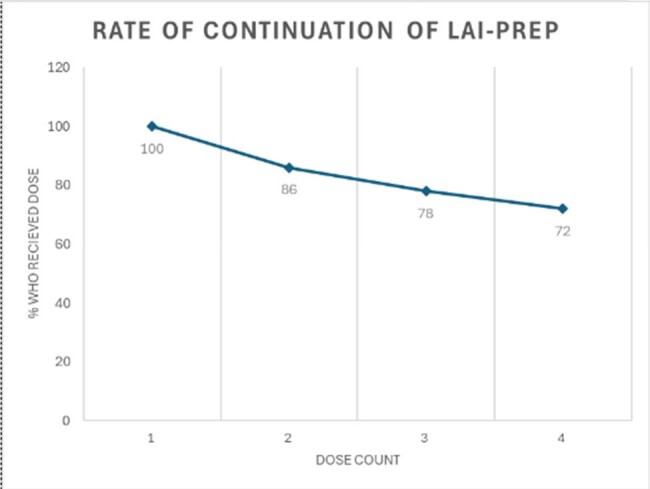

LAI-PrEP – long-acting injectable pre-exposure prophylaxis

**Results:**

During the 12-month period, 55 people initiated LAI-PrEP, with 36 eligible for the six-month PrEP persistence evaluation. This population was 83% men, 31% Black, 58% Hispanic/Latino, and the median age was 32 (range 20-58) years. 75% received oral PrEP previously. LAI-PrEP six-month persistence rate was 72% (26 of 36). Among the 10 people with LAI-PrEP discontinuation, 1 transitioned to oral PrEP, 2 no longer had an indication for PrEP (change in serodifferent relationship status), and 7 were lost to follow-up. LAI-PrEP persistence differed by race (46% Black vs 88% white, p=0.007) and sexual orientation (37% heterosexual, 89% gay, 67% bisexual, p=0.019).

**Figure:**
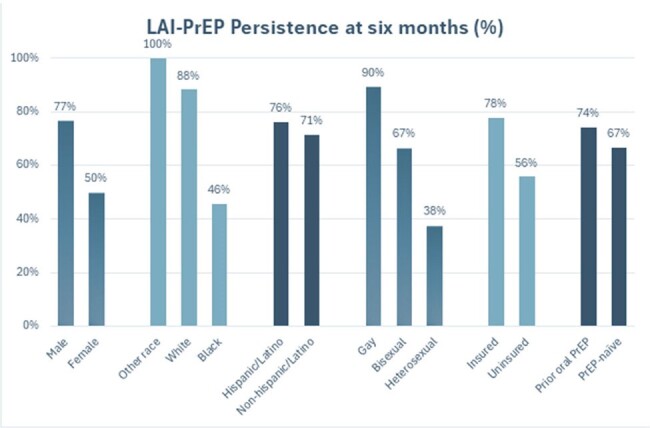

LAI-PrEP – long-acting injectable pre-exposure prophylaxis

**Conclusion:**

In the first year of LAI-PrEP implementation, LAI-PrEP uptake was favorable in our clinic, especially among priority populations for HIV prevention. Loss to follow-up was the most common reason for discontinuation, but change in PrEP method or loss of PrEP indication accounted for 30% of discontinuations. Nevertheless, the differences in persistence rates suggest a need for targeted interventions to improve long-term adherence in minority and heterosexual groups. Addressing PrEP persistence in these populations is crucial for optimizing the impact of LAI-PrEP in communities with a high burden of new HIV infections.

**Disclosures:**

**All Authors**: No reported disclosures

